# Sense of Coherence and Burnout in Healthcare Professionals in the COVID-19 Era

**DOI:** 10.3389/fpsyt.2021.709587

**Published:** 2021-08-02

**Authors:** Kristina Stoyanova, Drozdstoy Stoyanov Stoyanov

**Affiliations:** ^1^RIMU (Research Institute at Medical University of Plovdiv), Division of Translational neuroscience, Plovdiv, Bulgaria; ^2^Department of Psychiatry and Medical Psychology, Medical University of Plovdiv, Plovdiv, Bulgaria

**Keywords:** sense of coherence, salutogenic functioning, burnout, COVID-19, health care professionals, mentalization, Bulgaria

## Abstract

Emotional exhaustion in the context of vulnerability to burnout is a part of the universal narrative of high stress and systematically reported in healthcare professionals. The sense of coherence (SOC) is a salutogenic construct, operationalized by A. Antonovsky as a generalized resistance resource (GRR) to stress in three dimensions: meaningfulness (Me), the desire of a person to be motivated to cope; comprehensibility (C), the belief that the challenge is understood; and manageability (Ma), the belief that coping resources are available. The relation between SOC and the dimensions of burnout—Emotional Exhaustion (EE), Depersonalization (D), and Personal Accomplishment (PA)—is a part of salutogenic functioning, which reveals the inner motivation and self-organization of the psycho-emotional energetic resource. This study traces the salutogenic functioning of Bulgarian healthcare professionals during the pandemic. A general psychological background of coherence and exhaustion has been identified. All components of the SOC were positively correlated to Personal Accomplishment. Emotional Exhaustion and Depersonalization correlated negatively with coherence. SOC was validated as a possible determinant to predict the reduction of exhaustion and depersonalization as well as high levels of professional performance. The dimension of Meaningfulness in the coherence phenomenon was demonstrated to have the highest predictive value for professional burnout.

## Introduction

Various mental changes and issues, which have their own evolutionary significance, have been observed in the contemporary reality of worldwide crisis caused by the pandemic. Since the general human sense of normality has been disrupted, a number of questions concerning health and pathology have emerged as the natural consequence. Therefore, the origination of a novel concept termed *a new normality*, which is associated with COVID-19 in the public speech, is no coincidence (1–6). The pathogen itself has been denominated as SARS-CoV-2 (7, 8). The healthcare management worldwide has been challenged to transform its conventional operational and applied practical frameworks (9). Such biopsychosocial eventfulness is a major facilitator of professional burnout in all its phenomenological forms considering the various stress levels. Vulnerability to burnout is a compound construct consisting of diathesis (personality trait) and stress (psychological climate); hence, it is complementary to specific personality profiles. Dimensions of the professional environment such as autonomy, cohesion, trust, support, recognition, fairness, and innovation exhibit a negative correlation with EE. Furthermore, PA, or performance in the context of burnout, correlates positively with traits such as persistence, self-directedness, and self-transcendence [(10), p. 4–92].

The existing agenda of the world has been objectively deconstructed. From the perspective of analytical psychotherapy, COVID-19 is a catastrophic phenomenon, and the social living body has suffered an entropic collapse (11). The political decisions in the affected countries have been focused altogether on unified measures for prevention and control in accordance with the World Health Organization (WHO) guidelines (12). The measures consisted of quarantine, lockdown, social distancing, mandatory usage of face masks, working and tutoring in an online environment, establishment of hospital wards dedicated to the treatment of patients with COVID, and the development of therapeutic strategies and vaccines. This inevitable repertoire of critical readiness and coping in the field of traditional medicine has been defined as a focal point on pathology and disease, or pathogenic orientation by Antonovsky (13, 14). He designated the forces driving the different health attitudes as non-entropic factors and created the sense of coherence (SOC) scale. Even though this scale did not exhaust the entirety of salutogenic models, it remained as a central construct in his theory. The salutogenic idea affirmed the ability of a person to use external and internal resources in order to manage ubiquitous stressful situations. In Antonovsky's concept of SOC, there is a cognitive component represented by the dimension of comprehensibility, an instrumental component delineated by the manageability, and a motivational component assessed through the dimension of meaningfulness (13). According to this theoretical framework, the processes of entropy and negentropy in the collective human community require timely psychosocial identification. Thus, it is of significant importance to observe these processes in cohorts composed of professionals groups of people engaged in the core of urgency, generated by the *independent variable* COVID-19.

People who are most actively engaged in the crisis management caused by COVID-19 are healthcare professionals. According to most authors, they are the most affected group. Common mental health consequences, which have arisen in the current situation, such as depression, anxiety, panic, anger, confusion, ambivalence, and financial distress, have occurred in this societal cohort in the context of previous pandemics (15). For example, depression, anxiety, and post-traumatic stress disorder (PTSD) were demonstrated to be the most common mental disorders in healthcare professionals during the 2003 SARS and 2014 Ebola virus pandemics (16–18). New representative studies reported insomnia and distress (19, 20). Psychological resilience has also been associated with symptoms of depression and anxiety in this group (21, 22). The results from contemporary research in the scientific literature support the unequivocal necessity for prioritization of mental healthcare in medical professionals.

In order to develop a solution that informs the management and organizational policies during a pandemic, and in essence salutogenic, it is of paramount significance to conduct studies addressing the generalized resistance resources (GRRs) in relevant vulnerable groups. The observed collective grief has been incorporated into a hypothesis that recognizes the emotions that have ascended during the COVID-19 pandemic as very similar to bereavement, as in the case of losing a loved one. They have been present in the general population as a result of the loss of normalcy due to the various restricting measures adopted around the world. Furthermore, this phenomenon can be viewed as a precondition to a sense of emptiness and loss of meaning in life (23, 24). The meaning in life has been analyzed as a stable factor, which in concordance with other factors may function as a buffer against stress reactions in the pandemic context. In addition, the attainment of a sense of meaning in life has been associated with low levels of anxiety and emotional distress (25). In a prospective study of the relationship between psychopathological symptoms and SOC before and after the COVID-19 outbreak, Schäfer et al. (26) have established an inverse proportion ratio. Higher levels of SOC have been associated with a lower severity of PTSD symptoms (27). In the past year, a large variety of scientific research has reported an increased frequency of anxiety disorders, depression, PTSD, and other mental health crisis as a detrimental effect from the general psychological distress in the conditions of COVID-19.

Rajkumar (28) has outlined a conceptual salutogenic framework for a mental health approach that can integrate the principles of existential positive psychology and salutogenesis, which has been based on thoroughly reviewed previous experience and a critical analysis. The highlights of this approach are assessing the psychological distress in terms of the risk for auto- or allo-aggression, comprehending suffering outside of the conventional diagnostic criteria, interpreting the “story of the person,” determining the individual's current SOC, and deriving therapeutic meaning from suffering. Suffering has been defined as a state of severe distress associated with events that threaten human integrity and intactness (29). In its holistic salutogenic aspect, suffering has been presented as a potential positive source of learning that can contribute to the restoration of lost or threatened integrity (30, 31).

## The Current Study

Our research paradigm was motivated by the existential problems focused on the sense of meaning and suffering, and the psychological constructs we chose to measure have a significant conceptual overlap. The profound symptoms of burnout are universally expressed *via* indifference, anhedonia, and loss of meaning (10, 32). In theory, meaningfulness exhibits the same psychological structure as in the dimensions of the SOC. Such a research perspective, which in high extent informs construct validity characteristics, is designed to identify salutogenic functioning in a specific stress context in a specific sample. The SOC is not a construct associated with culture (13). This fact is particular with regard to possible modifications in the existing guidelines, recommendations, and policies for re-adaptation and mental healthcare. We expected a substantial association and prognosis of SOC both in line with burnout and in line with personal accomplishment as an aspect to mental health. The need for proactive research to identify GRR in this group is outlined (28, 30, 32, 33).

## Materials and Methods

The survey was intended to be short and, after a block of questions on demographic data two assessment tools, was combined—Maslach Burnout Inventory (MBI) with 22 items and a brief nine-item SOC scale (34, 35). The study was approved by the National Ethics Committee of the Bulgarian Association of Health Care Professionals with Protocol No. 2/10.05.2021.

### Measures of Burnout

Professional burnout is assessed in three dimensions: Emotional Exhaustion, Depersonalization, and Personal Accomplishment (the last ranking scale is reversed). The original Maslach Burnout scale is composed of 22 items, which are answered on a seven-grade scale from 0 (never) to 6 (always). Emotional Exhaustion (EE) expresses the depletion of psychoenergetic resources of human. Depersonalization (D) scale in this tool allows an assessment of withdrawal—emotionally, mentally, and socially—as well as a negative attitude toward oneself and others, without including psychopathology. In the Bulgarian standardization of the Maslach test, this scale is translated as Dehumanization. Dehumanization is analyzed as an alienation from human suffering. Personal Accomplishment (PA) is the dimension of personal effectiveness, assessment of interest in development, and improvement in the profession. In the Bulgarian adaptation, the scale is translated as reduced Personal Accomplishment (10). Cronbach's α for a summated score based on the 22 MBI items is 0.732.

### Sense of Coherence

We used the Norwegian version, a shortened nine-item version of Antonovsky's original 13-item SOC scale, which has very good psychometric indicators with epidemiological data from a mental health survey of adults in local communities (*N* = 1,062). The instrument has not been applied in Bulgaria. The stages of translation and cultural adaptation were as follows: translation with conceptual and linguistic evaluation, back-translation, comparison of the source and target version, and verification of the new instrument. Cronbach's α to assess the reliability of the SOC scale in our sample is 0.807. SOC is assessed in three components: Meaningfulness (Me), Manageability (Ma), and Comprehensibility (C). Antonovsky defines SOC as a global orientation, which expresses the extent to which a person has a pervasive, enduring, and dynamic feeling of confidence that the diversity of stimuli deriving from internal and external environments in the course of living is structured, predictable, explicable (the cognitive component—comprehensibility); the resources are available to meet the demands posed by these stimuli (the instrumental component—manageability); and these demands are challenges, worthy of investment and engagement (the motivational component—meaningfulness). Three of the items have been reversed. The original scale for each item is seven-grade: from 1 (very often) to 7 (very seldom or never). A high score is an indicator of high SOC (34).

### Participants

Participants in the present study are 147 Bulgarian healthcare professionals aged between 24 and 63 years. The sample is predominantly female: there are 146 women in it. The research was conducted anonymously, voluntarily, and online with the collaboration of the Bulgarian Association of Healthcare Professionals in Bulgaria 1 year after the announcement of national quarantine on March 13, 2021. In that time, the questionnaires were administered. All specialists live and work on the territory of Bulgaria: 15.6% (*N* = 23) in the capital, 76.2% (*N* = 112) in a district town, 7.5% (*N* = 11) in a small town, and 0.7% (one person) in a village. The duration of their professional experience varies from 1 to 43 years' work in the specialty. Of them report, 45.5% (61) that they combine more than one professional role: in 17.7% (26), this activity is student training, and 6.8% (10) of the respondents have a private medical practice. The rest of them work in two places in the healthcare system. The Google Form platform is used. All participants have declared an electronic informed consent to participation. American Psychological Association (APA) ethical guidelines for psychological research have been followed (36).

### Data Analysis

The research variables are described in terms of central tendency measures (mean, median, mode, and SDs) and data distribution (excess and asymmetry). Demographic and professional characteristics of the participants are also presented. Then reliability analysis of using scales was performed. Linear relation between the variables was tested with Pearson's correlation coefficient. Linear regression analysis was performed to assess the prognostic values of burnout dimensions from the SOC. SPSS 23.0 has been used for the data processing and analysis.

## Results

The distribution of responses of all items follows normal distribution—the coefficients of asymmetry (skewness) and excess (kurtosis) are in the interval (−1; 1). The mean age of health professionals is 46.4, and the mean duration of their professional experience is 22.7 years (Table 1).

**Table 1:** Descriptive statistics of the distributions with data for SOC and MBI subscales and other variables in the analyses.

	***N***	**Minimum**	**Maximum**	**Mean**	**Median**	**Mode**	**Std. deviation**	**Skewness (asymmetry)**	**Kurtosis (excess)**
EE	146	3	51	23.42	22.00	22	11.065	0.254	−0.608
D	147	0	22	7.96	7.00	6	5.391	0.519	−0.580
PA	147	17	42	31.21	31.00	30	5.410	−0.152	−0.232
Me	147	4	21	15.78	16.00	17	3.760	−0.632	−0.052
Ma	147	3	21	12.89	13.00	13	4.379	−0.361	−0.559
C	147	5	21	14.29	14.00	16	3.877	−0.065	−0.804
General SOC	147	15	62	42.96	43.00	35	9.826	−0.804	−0.516
Age (years)	146	24	63	46.39	47.00	52	9.135	−0.410	−0.098
PE	147	1	43	22.74	24.00	30	11.053	−0.377	−0.598

*PE, professional experience; SOC, sense of coherence; MBI, Maslach Burnout Inventory; EE, Emotional Exhaustion; D, Depersonalization; PA, Personal Accomplishment; Me, meaningfulness; Ma, manageability; C, comprehensibility*.

The sample is represented almost exclusively by female subjects: only one male subject took part in it. The majority of health professionals are employed as nurses-−59.9% (*N* = 88). The next subgroup in the sample is composed of midwives-−17.7% (*N* = 26). The other health professionals are distributed as follows: psychiatrists 2% (*N* = 3), trainees in psychiatry 0.7% (*N* = 1), surgery nurses 2% (*N* = 3), nurses in oncology 0.7% (*N* = 1), nurse in intensive care 0.7% (*N* = 1), nurse in psychiatry 0.7% (*N* = 1), school nurse 0.7% (*N* = 1), perfusionist 0.7% (*N* = 1), senior nurses 1.4% (*N* = 2), emergency nurse 0.7% (*N* = 1), radiology technician 1.4% (*N* = 2), rehabilitator 0.7% (*N* = 1), medical laboratory assistants 4.1% (*N* = 6), clinical laboratory assistant 0.7% (*N* = 1), histological laboratory assistant 0.7% (*N* = 1), public health and health management—management function 0.7% (*N* = 1), paramedics 1.4% (*N* = 2), and assistants and lecturers in medical universities 2% (*N* = 3).

The values of Cronbach's α coefficient for reliability of the scales and subscales of methods are determined for Emotional Exhaustion (nine items), α = 0.932; Depersonalization (five items), α = 0.769; Personal Accomplishment (eight items), α = 0.559; a summated score based on the 22 MBI items, α = 0.732; Meaningfulness (three items), α = 0.643; Manageability (three items), α = 0.690; Comprehensibility (three items), α = 0.612; and General SOC (a summated score based on the nine items), α = 0.807 (Table 2).

**Table 2:** Internal consistency (Cronbach's α) of the SOC and MBI subscales.

**Scales**	**No. of items**	**Cronbach's α**
Emotional exhaustion	9	0.932
Depersonalization	5	0.769
Personal Accomplishment	8	0.559
MBI	22	0.732
Meaningfulness	3	0.643
Manageability	3	0.690
Comprehensibility	3	0.612
General SOC	9	0.807

*SOC, sense of coherence; MBI, Maslach Burnout Inventory*.

Correlation analysis indicates that Emotional Exhaustion and Depersonalization are significantly and negatively associated with all dimensions of SOC. The analysis between Emotional Exhaustion and Meaningfulness (*r* = −0.572, *p* < 0.01), Manageability (*r* = −0.606, *p* < 0.01), Comprehensibility (*r* = −0.432, *p* < 0.01), and General SOC (*r* = −0.661, *p* < 0.01) shows that in the aspect of exhaustion, the cognitive, instrumental, and motivational components that comprise coherence are unstable. The analysis between Depersonalization and Meaningfulness (*r* = −0.546, *p* < 0.01), Manageability (*r* = −0.443, *p* < 0.01), Comprehensibility (*r* = −0.306, *p* < 0.01), and General SOC (*r* = −0.527, *p* < 0.01) reveals that emotional and social withdrawal in burnout are also associated with low levels of coherence. In terms of Personal Accomplishment, the dimension of effectiveness and the interest in self-improvement in burnout assessment, it correlates positively and strongly with the components of coherence: Meaningfulness (*r* = 0.557, *p* < 0.01), Manageability (*r* = 0.443, *p* < 0.01), Comprehensibility (*r* = 0.383, *p* < 0.01), and General SOC (*r* = 0.562, *p* < 0.01). These associations might be interpreted in the context of salutogenic functioning (Table 3).

**Table 3:** Correlations between SOC and MBI scales.

	**Meaningfulness**	**Manageability**	**Comprehensibility**	**General SOC**
Emotional exhaustion	−0.572^**^	−0.606^**^	−0.432^**^	−0.661^**^
Depersonalization	−0.546^**^	−0.443^**^	−0.306^**^	−0.527^**^
Personal accomplishment	0.557^**^	0.443^**^	0.383^**^	0.562^**^

*SOC, sense of coherence; MBI, Maslach Burnout Inventory*.

** *Correlation is significant at the 0.01 level (two-tailed)*.

The prerequisites for linear regression analysis are fulfilled. All SOC scales significantly predict the dimensions of burnout.

Meaningfulness predicts Emotional Exhaustion, *F*_(1, 144)_ = 70.054, *p* < 0.001. The value of the adjusted coefficient of determination (adjusted *R*^2^) is 0.32; accordingly, 32% of the variance of EE can be explained by Me. Meaningfulness predicts Depersonalization, *F*_(1, 145)_ = 61.541, *p* < 0.001. The value of the adjusted *R*^2^ is −0.29; accordingly, 29% of the variance of D can be explained by Me. The significant values are with a minus sign, which indicates inverse relation. It implies that Me significantly predicts the reduction of EE and D. Meaningfulness predicts Personal Accomplishment, *F*_(1, 145)_ = 65.229, *p* < 0.001. The value of the adjusted *R*^2^ is 0.31; accordingly, 31% of the variance of PA can be explained by Me. In this regression model, the motivational component of the SOC significantly predicts the professional performance.

Manageability predicts Emotional Exhaustion, *F*_(1, 144)_ = 83.509, *p* < 0.001. The value of the adjusted coefficient of determination (adjusted *R*^2^) is 0.36; consequently, 36% of the variance of EE can be explained by Ma. Manageability predicts Depersonalization, *F*_(1, 145)_ = 35.434, *p* < 0.001. The value of the adjusted coefficient of determination (adjusted *R*^2^) is 0.19; consequently, 19% of the variance of D can be explained by Ma. The significant values are with a minus sign, which indicates inverse relation: Ma significantly predicts the low levels of EE and D. Manageability predicts Personal Accomplishment, *F*_(1, 145)_ = 35.404, *p* < 0.001. The value of the adjusted *R*^2^ is 0.19; accordingly, 19% of the variance of PA can be explained by Ma. In this regression model, the instrumental component of the SOC significantly predicts the professional efficiency.

Comprehensibility predicts Emotional Exhaustion, *F*_(1, 144)_ = 32.961, *p* < 0.001. The value of the adjusted coefficient of determination (adjusted *R*^2^) is 0.18; therefore, 18% of the variance of EE can be explained by C. Comprehensibility predicts Depersonalization, *F*_(1, 145)_ = 15.031, *p* < 0.001. The value of the adjusted coefficient of determination (adjusted *R*^2^) is 0.09; therefore, 9% of the variance of D can be explained by C. The significant values are with a minus sign, which indicates inverse relation: C significantly predicts the low values of EE and D. Comprehensibility predicts Personal Accomplishment, *F*_(1, 145)_ = 24.986, *p* < 0.001. The value of the adjusted *R*^2^ is 0.14; therefore, 14% of the variance of PA can be explained by C. In this regression model, the cognitive component of the SOC significantly predicts high professional efficiency.

General SOC predicts Emotional Exhaustion, *F*_(1, 144)_ = 111.500, *p* < 0.001. The value of the adjusted coefficient of determination (adjusted *R*^2^) is 0.43; accordingly, 43% of the variance of EE can be explained by C. General SOC predicts Depersonalization, *F*_(1, 145)_ = 15.031, *p* < 0.001. The value of the adjusted coefficient of determination (adjusted *R*^2^) is 0.27; accordingly, 27% of the variance of D can be explained by General SOC. The significant values are with a minus sign, which indicates inverse relation. Or General SOC significantly predicts the low levels of EE and D. General SOC predicts Personal Accomplishment, *F*_(1, 145)_ = 66.892, *p* < 0.001. The value of the adjusted *R*^2^ is 0.31; accordingly, 31% of the variance of PA can be explained by General SOC. In this regression model, all components of the SOC significantly predict high professional performance (Table 4).

**Table 4:** Results of linear regression analysis of relation between SOC and burnout.

**Independent variable/predictor**	***R*^**2**^**	***B***	**β**	***t***	***p***
**4.1. Emotional exhaustion**					
Meaningfulness	0.323	−1.684	−0.72	−8.370	0.000
Manageability	0.63	−1.531	−0.06	−9.138	0.000
Comprehensibility	0.81	−1.232	−0.32	−5.741	0.000
General SOC	0.32	−0.44	−0.61	−10.559	0.000
**4.2. Depersonalization**					
Meaningfulness	−0.93	−0.83	−0.46	−7.845	0.000
Manageability	0.91	−0.46	−0.43	−5.953	0.000
Comprehensibility	0.88	−0.26	−0.06	−3.877	0.000
General SOC	0.73	−0.89	−0.27	−7.473	0.000
**4.3. Personal accomplishment**			
Meaningfulness	0.06	0.01	0.57	8.076	0.000
Manageability	0.91	0.47	0.43	5.651	0.000
Comprehensibility	0.41	0.45	0.83	4.999	0.000
General SOC	0.11	0.09	0.62	8.179	0.000

*SOC, sense of coherence*.

## Discussion

Emotional or psychological distress has an identical phenomenology with the symptoms of professional burnout. Burnout is predicted by complex factors of distress emerging from the realities of the current pandemic. There is agreement in common literature that health professionals are among the most affected groups, as their medical profiles and affiliation confront them instrumentally against the personalized and global threat called COVID-19. This study identifies the general psychological background of exhaustion and coherence through the assessment of SOC and burnout in a Bulgarian sample of healthcare professionals. The results replicate relations reported in the literature regarding the moderating and mediating role of SOC on mental (ill) health (26, 27, 34, 37–39) but differ in terms of the current context of pandemic crisis.

We found substantial associations between all components of the SOC and the burnout dimensions. Meaningfulness and General SOC are associated with high levels of Personal Accomplishment on the one hand, and Emotional exhaustion and Depersonalization are associated with low levels of SOC on the other. These trends set the background for more salutogenic functioning of health professionals. Linear regression analysis using the Enter method revealed significant regression models that combine all SOC and burnout variables. The variation of Emotional Exhaustion is significantly predicted in 32% by Meaningfulness, 36% by Manageability, 18% by Comprehensibility, and 43% by General SOC. The variation of Depersonalization is significantly predicted in 29% by Meaningfulness, 19% by Manageability, 9% by Comprehensibility, and 27% by General SOC. The variation of Personal Accomplishment is significantly predicted in 31% by Meaningfulness, 19% by Manageability, 14% by Comprehensibility, and 31% by General SOC. Therefore, the combination of motivational, instrumental, and cognitive components of SOC significantly predicts professional performance and burnout in healthcare specialists. The moderating role of SOC in regard to professional and emotional stress has been confirmed. However, these results require further analysis to compare data across centers, professional groups, and cultural contexts.

In line with previous studies (28, 30, 33), we have identified GRR in relevant vulnerable groups as a critical premise in designing mental health intervention programs. The concept of SOC can influence some psychotherapeutic modalities and psychological counseling approaches. A hybrid form of psychosocial support that allows for multidisciplinary professional intervention is likely to be effective in groups of health professionals, which may include the next steps.

- *Psychological evaluation* of the psychological climate, levels of burnout, psychosocial stress and distress (10, 40), SOC, and GRR accordingly co-produce a local model of vulnerability and resilience (41)^1^.
- *Local training* and counseling in groups of managers and healthcare employees for interactive discussion and assessment of suffering, SOC, GRR, and subjective mental phenomena experienced during pandemic, supervised by a multidisciplinary team.
- *Multidisciplinary framework can potentially benefit from mentalization-based treatment (MBT) strategies*, which share common constructs from SOC. Therapeutic competencies and skills of treatment based on mentalization require not knowing (essentially acceptance of uncertainty) genuine and inquisitive therapist stance, support and empathy, clarification, exploration, challenge, affect focus, and relationship (42, 43).

## Conclusion

This study has delivered clear evidence about the association of constructs and measures underlying SOC and GRR as determinants of burnout in healthcare professionals. In our perspective, a guideline informed by evaluation of those factors can motivate MBT as a flexible and innovative strategy for psychosocial support aiming at the healthcare specialists in the COVID-19 era.

## Data Availability Statement

The raw data supporting the conclusions of this article will be made available by the authors, without undue reservation.

## Ethics Statement

The studies involving human participants were reviewed and approved by National Ethics Commission of the Bulgarian Association of Health Care Professionals. The patients/participants provided their written informed consent to participate in this study.

## Author Contributions

The study design was created by KS. The implementation of the study and the data analysis were performed by KS and DS. KS wrote the original manuscript of the article. DS reviewed and edited the manuscript. All authors have a coordinated contribution and have approved the submission of the article.

## Conflict of Interest

The authors declare that the research was conducted in the absence of any commercial or financial relationships that could be construed as a potential conflict of interest. The handling editor declared a past collaboration with one of the authors DS.

## Publisher's Note

All claims expressed in this article are solely those of the authors and do not necessarily represent those of their affiliated organizations, or those of the publisher, the editors and the reviewers. Any product that may be evaluated in this article, or claim that may be made by its manufacturer, is not guaranteed or endorsed by the publisher.

## Footnotes

^1^Generalized resistance resources are biological, material, and psychosocial factors that make it easier for people to perceive their lives as consistent, structured, and understandable. Typical generalized resources are money, knowledge, experience, well-being, healthy habits, commitment, social support, cultural capital, intelligence, traditions, and vision for life. Access to them increases the opportunities to coping with life's challenges and creating coherent experiences, i.e., strengthening the SOC (13).

## References

[1] Kapoor R. Is there a postnormal time? From the illusion of normality to the design for a new normality. Futures. (2011) 43:216–20. 10.1016/j.futures.2010.10.012

[2] Lemke M. New normality? Z Politikwiss. (2018) 28:607–11. 10.1007/s41358-018-0167-7

[3] Mugnano S Carnelli F. A “new normality” for residents and tourists: how can a disaster become a tourist resource? In: Bellini N, Pasquinelli C, editors. Tourism in the City. (2017). p. 321–32.

[4] González-Sanguino C Ausín B Castellanos MA Saiz J Muñoz M. Mental health consequences of the Covid-19 outbreak in Spain. A longitudinal study of the alarm situation and return to the new normality. Prog Neuro Psychopharmacol Biol Psychiatry. (2021) 107:110219. 10.1016/j.pnpbp.2020.110219 33338556 PMC7833458

[5] Tesar M. Towards a post-covid-19 ‘New Normality?': physical and social distancing, the move to online and higher education. Policy Fut Educ. (2020) 18:556–9. 10.1177/1478210320935671

[6] Emiliani F Contarello A Brondi S Palareti L Passini S Romaioli D. Social representations of “Normality”: everyday life in old and new normalities with covid-19. Papers Soc Represent. (2020) 29:9.1–9.36. Available online at: https://psr.iscte-iul.pt/index.php/PSR/article/download/552/472

[7] WHO-Convened Global Study of Origins of SARS-CoV-2: China Part. Available online at: https://www.who.int/publications/i/item/who-convened-global-study-of-origins-of- sars-cov-2-china-part (accessed May 1, 2021).

[8] WHO Coronavirus (COVID-19) Dashboard. Available online at: https://www.who.int/redirect-pages/page/novel-coronavirus-(covid-19)-situation- dashboard (accessed May 1, 2021).

[9] Lega F Palumbo R. Leading through the ‘new normality' of health care. Health Serv Manag Res. (2021) 34:47–52. 10.1177/0951484820987496 33601958

[10] Stoyanov D. New Model of Burn Out Syndrome: Towards Early Diagnosis and Prevention. Aalborg: River Publishers (2014).

[11] Ferri G. ZERO TIME 2020: the Time of Limitations. [TEMPO ZERO 2020: il tempo del limite] (2020). Available online at: https://www.analisi-reichiana.it/wp-content/uploads/2020/04/TEMPO-ZERO-di- G.Ferri_.pdf (accessed March 5, 2021).

[12] WHO. Country & Technical Guidance - Coronavirus Disease (COVID-19). Available online at: https://www.who.int/emergencies/diseases/novel-coronavirus-2019/technical-guidance-publications

[13] Antonovsky A. Health promoting factors at work: the sense of coherence. In: Kalimo R, EI-Batawi MA, Coope CL, editors. Psychosocial Factors at Work and Their Relation to Health. Geneva: World Health Organization (1987). p. 153–67. Available online at: https://www.semanticscholar.org/paper/Psychosocial-factors-at-work-and-their- relation-to-Kalimo-El-Batawi/6103044878cbbb3b864a247a6068b7457a51336c (accessed May 1, 2020).

[14] Antonovsky A. The salutogenic model as a theory to guide health promotion. Health Promot Int. (1996) 11:11–8. 10.1093/heapro/11.1.11

[15] Black Dog Institute. Mental Health Ramifications of COVID-19: The Australian Context. (2020). Available online at: https://blackdoginstitute.org.au/docs/default-source/default-document- library/20200319_covid19-evidence-and-reccomendations.pdf (accessed May 10, 2021).

[16] Dong L Bouey J. Public mental health crisis during COVID-19 pandemic, China. Emerg Infect Dis. (2020) 26:1616–8. 10.3201/eid2607.200407 32202993 PMC7323564

[17] Maunder R Lancee W Balderson K Bennett J Borgundvaag B Evans S. Long-term psychological and occupational effects of providing hospital healthcare during SARS outbreak. Emerg Infect Dis. (2006) 12:1924–32. 10.3201/eid1212.060584 17326946 PMC3291360

[18] Tam CW Pang EP Lam LC Chiu HF. Severe acute respiratory syndrome (SARS) in Hong Kong in 2003: stress and psychological impact among frontline healthcare workers. Psychol Med. (2004) 34:1197–204. 10.1017/S0033291704002247 15697046

[19] Arora T Grey I Ostlundh L Lam KBH Omar OM Arnone D. The prevalence of psychological consequences of COVID-19: a systematic review and meta-analysis of observational studies. J Health Psychol. (2020) 1–20. 10.1177/1359105320966639. [Epub ahead of print]. 33118376

[20] Liu S Yang L Zhang C Xiang YT Liu Z Hu S. Online mental health services in China during the COVID-19 outbreak. Lancet Psychiatry. (2020) 7:e17–8. 10.1016/S2215-0366(20)30077-8 32085841 PMC7129099

[21] Di Tella M Romeo A Benfante A Castelli L. Mental health of healthcare workers during the COVID-19 pandemic in Italy. J Eval Clini Pract. (2020) 26:1583–7. 10.22541/au.158878917.77777713 32710481

[22] Foureur M Besley K Burton G Yu N Crisp J. Enhancing the resilience of nurses and midwives: pilot of a mindfulnessbased program for increased health, sense of coherence and decreased depression, anxiety and stress. Contemp Nurse. (2013) 45:114–25. 10.5172/conu.2013.45.1.114 24099232

[23] Berinato S. That Discomfort You're Feeling Is Grief. Harvard Business Review (2020). Available online at: https://hbr.org/2020/03/that-discomfort-youre-feeling-is-grief (accessed May 9, 2021).

[24] Taha N. Veel stress en angst om coronacrisis: ‘Mensen ervaren emoties die sterk lijken op rouw'. Algemeen Dagblad (2020). Available online at: https://www.ad.nl/binnenland/veel-stress-en-angst-om-coronacrisis-mensen-ervaren-emoties-die-sterk-lijken-op-rouw~a306b3f2/?referrer=https://www.google.com/ (accessed May 14, 2020).

[25] Trzebiński J Cabański M Czarnecka JZ. Reaction to the COVID-19 pandemic: the influence of meaning in life, life satisfaction, and assumptions on world orderliness and positivity. J Loss Trauma. (2020) 25:544–77. 10.1080/15325024.2020.1765098

[26] Schäfer SK Sopp MR Schanz CG Staginnus M Göritz AS Michael T. Impact of Covid-19 on public mental health and the buffering effect of sense of coherence. Psychother Psychosomat. (2020) 89:386–92. 10.1159/000510752 32810855 PMC7490493

[27] Schäfer SK Becker N King L Horsch A Michael T. The relationship between sense of coherence and post-traumatic stress: a meta-analysis. Eur J Psychotraumatol. (2019) 10:1562839. 10.1080/20008198.2018.1562839 30693079 PMC6338278

[28] Rajkumar RP. Suffering and salutogenesis: a conceptual analysis of lessons for psychiatry from existential positive psychology (PP2.0) in the setting of the COVID- 19 pandemic. Front Psychol. (2021) 12:646334. 10.3389/fpsyg.2021.646334 33897551 PMC8064119

[29] Cassell E. The Nature of Suffering and the Goals of Medicine. Oxford: Oxford University Press (2004).

[30] Oliveira CC. Suffering and salutogenesis. Health Promot Int. (2015) 30:222–7. 10.1093/heapro/dau061 25073763

[31] Cuellar ET Zaiontz RG. Salutogenic nursing education: a summative review. J Nurs Educ Pract. (2003) 3:89–101. 10.5430/jnep.v3n5p89

[32] Stoyanov D Stoikova M Tornyova B Tilov B Turnovska T Mateva N. Burn out syndrome in health care personnel: ethical, psychological and methodological implications. In: 5th Balkan Congress on the History and Ethics Medicine 11-15. X. 2011, Istanbul (2011). p. 449–62.

[33] Fekete OR Kinn LG Larsen TMB Langeland E. Salutogenesis as a theoretical framework for psychosocial rehabilitation: the case of the Clubhouse model. Int J Qual Stud Health Well-being. (2020) 15:1748942. 10.1080/17482631.2020.1748942 32249690 PMC7170324

[34] Klepp OM Mastekaasa A Sorensen T Sandanger I Kleiner R. Structure analysis of Antonovsky's sense of coherence from an epidemiological mental health survey with a brief nine-item sense of coherence scale. Int J Methods Psychiatr Res. (2007) 16:11–22. 10.1002/mpr.197 17425244 PMC6878461

[35] Maslach C Jackson SE Leiter M. The maslach burnout inventory manual. In: Zalaquett CP, Wood RJ, editors. Evaluating Stress: A Book of Resources. Publisher: The Scarecrow Press (1997). Available online at: https://www.researchgate.net/publication/277816643_The_Maslach_Burnout_Inventor y_Manual (accessed August 2, 2017).

[36] APA. Ethical Principles of Psychologists and Code of Conduct. Available online at: https://www.apa.org/ethics/code/ (accessed May 8, 2021).

[37] Barni D Danioni F Canzi E Ferrari L Ranieri S Lanz M. Facing the COVID-19 pandemic: the role of sense of coherence. Front Psychol. (2020) 11:578440. 10.3389/fpsyg.2020.578440 33240166 PMC7677188

[38] Cilliers F Kossuth S. The relationship between organisational climate and salutogenic functioning. SA J Indust Psychol. (2002) 28:8–13. 10.4102/sajip.v28i1.42

[39] Cilliers F. Burnout and Salutogenic Functioning of Nurses. (2003). Available online at: http://uir.unisa.ac.za/bitstream/handle/10500/14575/burnout%20and%20salutogenic.p df?sequence=1 (accessed January 2, 2019). 10.4102/curationis.v26i1.1296 14509120

[40] Antonovsky A. Health, Stress and Coping. Washington: Jossey-Bass (1979).

[41] Stoyanova K Stoyanov D. Burn out accross persons and systems: comparative studies on vulnerability and resilience. Eur J Person Centered Healthcare. (2019) 7:410–1. 10.5750/ejpch.v7i2.1744

[42] Bateman W Bales D Hutsebaut J. A Quality Manual for MBT. (2014). Available online at: https://www.annafreud.org/media/1217/a-quality-manual-for-mbt-edited-april-23rd- 2014-2.pdf (accessed May 10, 2021).

[43] Matanova V Kolev M Tohme P Kostova Z. Brain-Based Treatment. New York: Nova Science Publishers (2020).

